# Circulation of four *Anaplasma phagocytophilum* ecotypes in Europe

**DOI:** 10.1186/1756-3305-7-365

**Published:** 2014-08-15

**Authors:** Setareh Jahfari, E Claudia Coipan, Manoj Fonville, Arieke Docters van Leeuwen, Paul Hengeveld, Dieter Heylen, Paul Heyman, Cees van Maanen, Catherine M Butler, Gábor Földvári, Sándor Szekeres, Gilian van Duijvendijk, Wesley Tack, Jolianne M Rijks, Joke van der Giessen, Willem Takken, Sipke E van Wieren, Katsuhisa Takumi, Hein Sprong

**Affiliations:** Laboratory for Zoonoses and Environmental Microbiology, National Institute for Public Health and Environment (RIVM), Antonie van Leeuwenhoeklaan 9, P.O. Box 1, Bilthoven, The Netherlands; Evolutionary Ecology Group, University of Antwerp, Antwerp, Belgium; Research Laboratory for Vector Borne Diseases, Queen Astrid Military Hospital, Brussels, Belgium; Animal Health Service Deventer, Deventer, The Netherlands; School of Veterinary Medicine, St. George’s University, True Blue, West indies, Grenada; Department of Parasitology and Zoology, Faculty of Veterinary Science, Szent Istvan University, Budapest, Hungary; Laboratory of Forestry, Department of Forest and Water Management, Ghent University, Ghent, Belgium; Dutch Wildlife Health Centre, Utrecht University, Utrecht, The Netherlands; Laboratory of Entomology, Wageningen University, Wageningen, The Netherlands; Resource Ecology Group, Wageningen University, Wageningen, The Netherlands

**Keywords:** *Anaplasma phagocytophilum*, Zoonoses, *Ixodes ricinus*, Wildlife, Epidemiology

## Abstract

**Background:**

*Anaplasma phagocytophilum* is the etiological agent of granulocytic anaplasmosis in humans and animals. Wild animals and ticks play key roles in the enzootic cycles of the pathogen. Potential ecotypes of *A. phagocytophilum* have been characterized genetically, but their host range, zoonotic potential and transmission dynamics has only incompletely been resolved.

**Methods:**

The presence of *A. phagocytophilum* DNA was determined in more than 6000 ixodid ticks collected from the vegetation and wildlife, in 289 tissue samples from wild and domestic animals, and 69 keds collected from deer, originating from various geographic locations in The Netherlands and Belgium. From the qPCR-positive lysates, a fragment of the *groEL*-gene was amplified and sequenced. Additional *groEL* sequences from ticks and animals from Europe were obtained from GenBank, and sequences from human cases were obtained through literature searches. Statistical analyses were performed to identify *A. phagocytophilum* ecotypes, to assess their host range and their zoonotic potential. The population dynamics of *A. phagocytophilum* ecotypes was investigated using population genetic analyses.

**Results:**

DNA of *A. phagocytophilum* was present in all stages of questing and feeding *Ixodes ricinus,* feeding *I. hexagonus, I. frontalis*, *I. trianguliceps,* and deer keds, but was absent in questing *I. arboricola* and *Dermacentor reticulatus.* DNA of *A. phagocytophilum* was present in feeding ticks and tissues from many vertebrates, including roe deer, mouflon, red foxes, wild boar, sheep and hedgehogs but was rarely found in rodents and birds and was absent in badgers and lizards. Four geographically dispersed *A. phagocytophilum* ecotypes were identified, that had significantly different host ranges. All sequences from human cases belonged to only one of these ecotypes. Based on population genetic parameters, the potentially zoonotic ecotype showed significant expansion.

**Conclusion:**

Four ecotypes of *A. phagocytophilum* with differential enzootic cycles were identified. So far, all human cases clustered in only one of these ecotypes. The zoonotic ecotype has the broadest range of wildlife hosts. The expansion of the zoonotic *A. phagocytophilum* ecotype indicates a recent increase of the acarological risk of exposure of humans and animals.

**Electronic supplementary material:**

The online version of this article (doi:10.1186/1756-3305-7-365) contains supplementary material, which is available to authorized users.

## Background

*Anaplasma phagocytophilum* is an obligate intracellular bacterium of the family *Anaplasmataceae* in the order Rickettsiales that causes disease in humans and animals [1]. It infects neutrophils, resulting in influenza-like symptoms clinically and on rare occasions is even a fatal condition in humans [2]. The first cases of Human Granulocytic Anaplasmosis (HGA) were reported from the USA in 1994 [3]. This incidence has increased gradually to 6.1 cases per million persons in 2010. The first European case was reported from Slovenia in 1995. Since, HGA cases have been occasionally reported throughout Europe [4]. It is unclear to what extent HGA poses a risk to public health in Europe: epidemiological data on the disease incidence and disease burden is either incomplete or lacking from most European countries [5]. The non-specificity of the reported symptoms, poor diagnostic tools and lack of awareness of public health professionals further complicate these estimations [4, 6, 7].

*Anaplasma phagocytophilum* is transmitted to humans by the bite of an infected tick [8]. The main vector in Europe is *I. ricinus,* which also transmits *Borrelia burgdorferi* sensu lato, the causative agent of Lyme borreliosis. In the Netherlands, Lyme borreliosis is on the rise: there has been a threefold increase in consultations of general practitioners for tick bites and Lyme borreliosis since 1994. This rise can be partially explained by spatiotemporal increases in the abundance and activity of questing ticks [9–11]. It is to be expected that growth in tick abundance and activity will also increase the risk of human exposure to other tick-borne pathogens such as *A. phagocytophilum*, but evidence for this extrapolation is lacking [12, 13].

*Anaplasma phagocytophilum* is maintained in nature through enzootic cycles between ticks and wild animals [8]. The pathogen has been detected in ticks in most European countries and the infection rates range from 0.4% to 67% [4]. In the Netherlands, infection rates in questing nymphs and adults vary between 0% and 8% [12]. *Anaplasma phagocytophilum* has been detected in a wide range of wildlife species, including ruminants, rodents, insectivores, carnivores, birds and even reptiles [4]. The relative roles of each tick stage and wildlife species in the enzootic life cycles of *A. phagocytophilum* have not been fully elucidated yet [4].

*Anaplasma phagocytophilum* is currently considered as a single bacterial species. Cross-infection experiments, where isolates from distinct host origins were not uniformly infectious for heterologous hosts, indicate that host specialization of *A. phagocytophilum* may occur [14, 15]. Furthermore, *Anaplasma phagocytophilum* can be genetically divided into either a few or many subclusters, depending on the genetic markers used. As sequence-based clusters in the bacterial world appear to correspond to an “ecotype”, defined as a population of cells in the same ecological niche [16], subclustering may be related to variation in space, host preference, and pathogenicity. Initially, sequences from the 16S rRNA gene have been used for subclustering, but this gene was shown to not be informative enough to delineate distinct ecotypes of *A. phagocytophilum* [17, 18]. Highly variable gene fragments encoding for major surface proteins [19, 20], and *ankA*, a secretory protein [21–23], have been used as well. The *groEL* heat shock operon has an intermediate genetic variability and is expected to act as a marker for demographic analyses [24–27]. Sequences from the *groEL* operon have been shown to more clearly delineate ecotypes of *A. phagocytophilum* than do sequences of the 16S rRNA gene [24, 28].

Knowledge on the distribution of *A. phagocytophilum* in ticks and wildlife in the Netherlands and Belgium is scarce. The aim of this study was to investigate the distribution of *A. phagocytophilum* in different stages of endemic tick species and in wildlife hosts and free ranging domestic animals. The potential vectors and animal samples were tested by qPCR and conventional PCR, to determine whether they were infected with *A. phagocytophilum*. We investigated whether genetic delineation, based on *groEL*, correlates with host distribution/species and zoonotic potential. To assess whether the differential distribution of the genetic variants was due to geographic variation, all available *groEL* sequences of European *A. phagocytophilum* isolates were collected and subjected to similar analyses. Population genetic analyses were used to determine which of the ecotypes is expanding.

## Methods

### Collection of samples and DNA extraction

Questing *I. ricinus* and *Dermacentor reticulatus* were collected by blanket dragging at 17 different sites in The Netherlands and Belgium [29, 30]. *Ixodes arboricola* and *I. frontalis* (nymphs and adults and fed larvae) were collected from bird nest boxes and from birds that were captured with mistnets and nest traps in forested areas around the city of Antwerp (Belgium) [30]. *Ixodes hexagonus* feeding on European hedgehogs (*Erinaceus europaeus*) were collected in a hedgehog-shelter [29]. *Ixodes trianguliceps and I. ricinus* feeding on bank voles (*Myodes glareolus)* and wood mice (*Apodemus sylvaticus*) were collected at several different sites in The Netherlands and Belgium. *Ixodes ricinus* feeding on red deer (*Cervus elaphus*), European mouflon (*Ovis orientalis musimon*), wild boar (*Sus scrofa*), sheep (*Ovis aries*), wood mouse (*A. sylvaticus*), and sand lizard (*Lacerta agilis*) has been described in previous studies [29, 31, 32]. *Ixodes ricinus* feeding on roe deer (*Capreolus capreolus*) were collected at various localities by volunteers from deer-shelters. Deer keds (*Lipoptena cervi*) were collected from culled roe deer by hunters. Spleen samples were obtained from 19 animal species. These included samples from roe deer, several bird species and badgers (*Meles meles*), which were found dead or were euthanized and sent to the Dutch Wildlife Health Centre for postmortem examination. The spleen samples obtained from foxes (*Vulpes vulpes*) as well as the capture of wild rodents have been described elsewhere [29, 33]. EDTA-blood from clinically and laboratory confirmed anaplasmosis from horses were collected in a veterinary hospital [34]. DNA from questing ticks was extracted by alkaline lysis [32]. Blood and spleen samples were kept frozen (−80°C) until testing. DNA from engorged ticks, deer keds, and tissue samples was extracted using the Qiagen DNeasy Blood & Tissue Kit [29].

### Polymerase chain reactions and sequencing

All samples were screened for the presence of *A. phagocytophilum* DNA with a real-time polymerase chain reaction (qPCR) targeting a 77-bp portion of the *msp2* gene. The primers used were ApMSP2F (5’-atggaaggtagtgttggttatggtatt-‘3) and ApMSP2R (5’-ttggtcttgaagcgctcgta-‘3), and the probe was ApMSP2P (5’-tggtgccagggttgagcttgagattg-‘3) labeled with FAM6 [35]. This qPCR was performed in a multiplex format with *Neoehrlichia mikurensis* [29]. qPCR-positive samples were analysed further with primers targeting a fragment of the *groEL* gene of *A. phagocytophilum* [36]. All sequences were confirmed by sequencing both strands. The sequences were stored and analysed in Bionumerics (Version 7.1, Applied Math, Belgium), after subtraction of the primer sequences.

### Molecular epidemiological database

*Anaplasma phagocytophilum groEL* DNA sequences with the geographical origin (country) and the host species from which the isolate originated were also downloaded from the Entrez Nucleotide Database (GenBank, NCBI). *Anaplasma phagocytophilum* sequences originating from Northern white-breasted hedgehogs (*Erinaceus roumanicus*) were from a previous study [37]. Sequences that did not originate from natural isolates were excluded. Sequences that were too short to cover regions of variation were also excluded from further analysis. A literature search was performed to specifically extract *A. phagocytophilum groEL* DNA sequences from human patients in Europe [38–43]. DNA sequences and epidemiological data used for this study are given in the Additional file 1: Table S1.

### Phylogenetic and population genetic analysis

We delineated four *A. phagocytophilum* clusters (called ecotypes) by visually inspecting a phylogenetic tree (Additional file 2: Figure S1). A best-scoring maximum likelihood tree was obtained using RAxML 7.5.5 [44] with the option rapid bootstraps (n = 100). Each codon-position was separately analysed using a general-time-reversible model of base substitutions, gamma-distributed rates and invariant proportions. These models of DNA evolution were determined using PartitionFinder 1.0.1 [45]. The sequences were aligned using MAFFT [46] with default options. Alignment was trimmed (position 642 to 1084) to exclude short sequences to visualize genealogy of *A. phagocytophilum* haplotypes using Haploviewer (http://www.cibiv.at/~greg/haploviewer). Population genetics measures (Ewens-Watterson test, Tajima’s D, Fu’s Fs) were calculated using Arlequin [47] using untrimmed alignment.

### Host distributions between and within ecotypes

One *A. phagocytophilum* ecotype might be over-represented among lysates from a particular host species. We tested this possibility using a multinomial model in which a lysate from a particular host species is evenly associated across all four ecotypes, i.e. with the probability of 1/4 per ecotype. We then estimated by the Monte Carlo method the probability that the number of most numerous ecotypes in a random realization from the multinomial is equal to or greater than the observed maximum among our lysates The probability (i.e. P-values) less than 0.05 were considered significant support for selective distribution. Counting distinct host species is an alternative measure of host diversity per ecotype. However, observed number of distinct host species is best avoided because the sample availability varied by ecotype and a straightforward comparison in this case is invalid. Therefore, we applied the rarefaction analysis to our datasets and calculated whether the differences in observed number of distinct host species per ecotype were statistically significant and not a random variation due to the sampling bias. For this purpose, we computed p-values using EstimateS (Version 9, R. K. Colwell, http://purl.oclc.org/estimates).

## Results

A total of 3493 questing nymphs and adult *I. ricinus* from various geographical areas in the Netherlands and Belgium were tested for the presence of *A. phagocytophilum* by PCR. DNA of *A. phagocytophilum* was found in 2.6% of the tested ticks (90/3493), and in 13 of the 17 investigated areas (Table 1). *Anaplasma phagocytophilum* DNA was also detected in 1.3% (5/386) questing *I. ricinus* larvae (Table 2). The infection rate of adult *I. ricinus* was significantly higher than that of larvae or nymphs. No significant difference was observed between the infection rates of larvae and nymphs (Table 2). *Anaplasma phagocytophilum* DNA was not detected in *I. arboricola* (n = 79) and *I. frontalis* (n = 13) collected from nest boxes, nor in questing *Dermacentor reticulatus* (n = 59), but was found in 42% of the deer keds (29/69) feeding on 10 roe deer.

**Table 1:** **Infection rates of** ***A. phagocytophilum*** **in questing** ***I. ricinus*** **nymphs and adults**

Location	Tested (n)	Positive (n)	Infection rate (CI)	
**Denekamp**	104	0	0.0%	(<2.8%)
**Vlaanderen-area (Belgium)**	114	0	0.0%	(<2.6%)
**Pyramide van Austerlitz**	270	1	0.3%	(<1.8%)
**Vijlenerbos**	328	1	0.3%	(0–1.5%)
**Kop van Schouwen**	238	2	0.8%	(0.1-2.7%)
Rijk van Nijmegen	53	0	0.0%	(<5.5%)
Ulvenhoutse bos	61	0	0.0%	(<36%)
Wallonië-area (Belgium)	106	1	0.9%	(0-5%)
Dintelse Gorzen	122	2	1.6%	(0.2-5.8%)
Duin en Kruidberg	457	8	1.8%	(0.8-3.4%)
Boswachterij Hardenberg	90	2	2.2%	(0.3-7.8%)
Dwingeloo-area	1071	35	3.3%	(2.3-4.5%)
Drents-Friese Wold	29	2	6.9%	(0.8-22%)
***Hoog Soeren***	*217*	*14*	*6.5%*	*(3.7-10.3%)*
***Brussels-area (Belgium)***	*153*	*10*	*6.5%*	*(3.1-11.7%)*
***Vrouwenpolder***	*86*	*7*	*8.0%*	*(3.3-16%)*
***Hoge Veluwe***	*47*	*5*	*10.6%*	*(4.0-22%)*
***Total of all ticks***	**3493**	**90**	**2.5%**	**(2.0-3.1%)**

Ticks were collected by blanket dragging on various locations in The Netherlands and Belgium (three locations). The 95%-confidence intervals, which were calculated using Fisher's exact test, are between brackets. The five locations with infection rates significantly lower than 3% are indicated in **bold**. The four locations with infection rates significantly higher than 3% are indicated in ***cursive bold***.

**Table 2:** **Infection rates of** ***A. phagocytophilum*** **in questing** ***I. ricinus*** **, divided by life stage**

Stage	Tested (n)	Positive (n)	Infection rate (CI)	
Larvae	386	5	1.3%	(0.4-3.0%)
Nymph	3090	68	2.2%	(1.7-2.8%)
Adult	306	18	5.9%	(3.5-9.1%)
*Female*	*113*	*5*	*4.4%*	*(1.5-10.2%)*
*Male*	*193*	*13*	*6.7%*	*(3.6-11.2%)*

The 95%-confidence intervals, which were calculated using Fisher's exact test, are between brackets. The infection rate of adults is significantly higher than larvae or nymphs (p < 0.05).

To investigate the possible vertebrate host species for *A. phagocytophilum*, tissue samples of many different animals were tested (Table 3). Spleen samples from roe deer (26/38), red foxes (8/81), one wood mouse (1/23) and one common black bird (1/11) were positive (Table 3). Other organs, except brain, of the wood mouse and common black bird were also tested positive in the *A. phagocytophilum* qPCR (data not shown). *Anaplasma phagocytophilum* DNA was also amplified from 14 clinically- and laboratory confirmed horses. No *A. phagocytophilum* DNA was detected in the spleen samples of other rodents (n = 45), insectivores (n = 11), songbirds (n = 26), and badgers (n = 40).

**Table 3:** **Presence of** ***A. phagocytophilum*** **in vertebrate tissue samples**

Species	Common name	Tested (n)	Positive (n)
*Apodemus flavicollis*	Yellow-necked mouse	2	0
***Apodemus sylvaticus***	**Wood mouse**	**23**	**1**
*Crocidura russula*	White-toothed shrew	5	0
*Microtus arvalis*	Common vole	8	0
*Myodes glareolus*	Bank vole	35	0
*Sorex araneus*	Common shrew	6	0
*Carduelis chloris*	Greenfinch	4	0
*Coccothraustes coccothraustes*	Hawfinch	2	0
*Fringilla coelebs*	Common chaffinch	3	0
*Parus major*	Great tit	4	0
*Phylloscopus trochilus*	Willow warbler	1	0
*Pyrrhula pyrrhula*	Bullfinch	1	0
*Turdus iliacus*	Redwing	5	0
***Turdus merula***	**Common blackbird**	**11**	**1**
*Turdus philomelos*	Song thrush	6	0
***Capreolus capreolus***	**Roe deer**	**38**	**26**
**Equus ferus caballus**	**Domestic horse**	**14**	**14**
***Vulpes vulpes***	**Red fox**	**81**	**8**
*Meles meles*	Badger	40	0
**Total**		**289**	**50**

DNA extracts from spleen and EDTA-blood of wildlife and horses were tested by qPCR. The presence of *A. phagocytophilum* was confirmed in most cases by conventional PCR using *groEL* specific primer pairs, followed by sequencing. Positive animal species are shown in bold.

Due to their protected status in the Netherlands and Belgium, it is very difficult to address the infection rate of *A. phagocytophilum* in wildlife. As a proxy for their infection rates, ticks feeding on wildlife were collected and tested. The infection rates of *I. ricinus* feeding on roe deer, red deer, hedgehog, sheep, and mouflon (Table 4) were significantly higher than the infection rate of questing adult *I. ricinus* (Table 2). Ticks from wild boar were also positive (5/48), but not significantly more than *I. ricinus* from the vegetation (Table 1). Only one of the 109 *I. ricinus* larvae feeding on wood mice were positive for *A. phagocytophilum*. This same wood mouse carried 18 *A. phagocytophilum-*negative larvae (data not shown). Only nine *I. trianguliceps* feeding on four wood mice (n = 4) and three bank voles (n = 5) were collected. All eight larvae were negative, whereas one female *I. trianguliceps* feeding on a wood mouse was *A. phagocytophilum*-positive (Table 4). Both *I. ricinus* (11/117) and *I. frontalis* (4/7) feeding on common black birds were *A. phagocytophilum*-positive (Table 4). One *I. frontalis* (1/194) and none of the *I. arboricola* feeding on great/blue tit were *A. phagocytophilum*-positive. *Ixodes ricinus* ticks feeding on sand lizards were all negative for *A. phagocytophilum* (Table 4).

**Table 4:** **Infection rates of** ***A. phagocytophilum*** **in different Ixodid species feeding on wildlife**

Ticks from	Ticks species tested (n)		Tick stage	Ticks positive (n)	Infection rate ticks (%)	Animals tested (n)	Animals with positive ticks (n)
*Apodemus sylvaticus*	IR	109	L	1	0.9% (0-5%)	26	1
*Apodemus sylvaticus*	IT	4	A/L	1	25% (1-81%)	4	1
*Myodes glareolus*	IT	5	L	0	0% (<52%)	5	0
*Turdus merula*	IR	117	N/L	11	9% (5-16%)	42	6
*Turdus merula*	IF	7	N	4	57% (18-90%)	6	3
*Parus major/caeruleus*	IF	194	A/N/L	1	1% (<3%)	120	3
*Parus major/caeruleus*	IA	13	A/N/L	0	0% (<25%)	13	0
*Lacerta agilis*	IR	165	A/N/L	0	0% (<2%)	93	0
*Sus scrofa*	IR	48	N	5	10% (3.5-23%)	8	4
***Erinaceus europaeus***	IH	193	A/N	44	23% (17-29%)	ND	ND
***Ovis orientalis musimon***	IR	233	A	120	52% (45-58%)	18	18
***Ovis aries***	IR	264	A	173	66% (59-71%)	24	24
***Capreolus capreolus***	IR	301	A/N/L	245	81% (77-86%)	38	35
***Cervus elaphus***	IR	409	A/N	351	86% (82-89%)	16	16
***Total***		**2062**		**956**		**413**	**111**

Larval (L), nymphal (N) and adult (A) stages of *Ixodes ricinus* (IR)*, I. trianguliceps* (IT), *I. frontalis* (IF), *I. arboricola* (IA) *and I. hexagonus* (IH) feeding on different vertebrate species were tested for the presence of *A. phagocytophilum* DNA. The infection rates of ticks from animal species in **bold** are significantly higher than those of ticks from the vegetation (Table 1). The 95%-confidence intervals of these infection rates, which were calculated using Fisher's exact test, are between brackets. Data from sand lizards are derived from a previous study [32].

**Figure 1:**
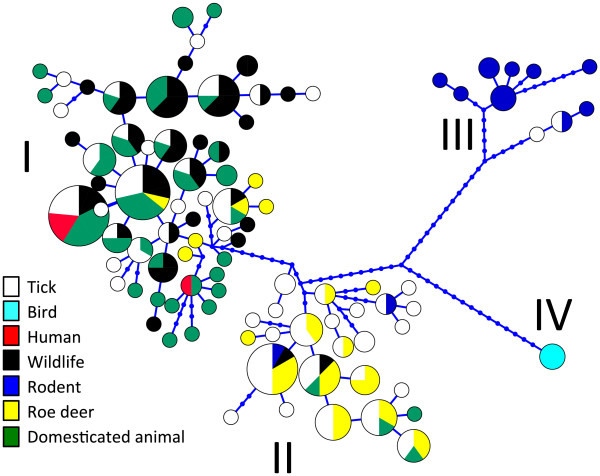
**Genealogy of** ***A. phagocytophilum*** **haplotypes.** Only 228 isolates of out of 548, representing all 97 haplotypes are shown. Of each haplotype, only one isolate per host from each country is used. An open circle is a haplotype. It is colored by the isolation origin (host species) and drawn in proportion to the sample size. A small blue dot is a missing haplotype. A blue edge is a mutation. The haplotype genealogies were made using Haploviewer software [48]. Roman numerals label the four ecotypes, which were inferred from a phylogenetic tree (Additional file 2: Figure S1).

In total, 162 *groEL* sequences were obtained from the qPCR-positive samples. Together with the *groEL* sequences from Genbank a phylogenetic tree was reconstructed (Additional file 2: Figure S1). Four major *A. phagocytophilum* clusters, called ecotypes, could clearly be delineated from this tree. Bootstrap support values were: 98% (ecotype I), 100% (ecotype III) and 100% (ecotype IV). Bootstrap support values for ecotype II was not explicitly computed, but the high support values for the ecotypes I, III and IV imply that the ecotype II is similarly well supported. These four ecotypes were also visually distinguishable in haplotype genealogies in samples both from all over Europe (Figure 1) and from the Netherlands/Belgium (not shown). Based on these four ecotypes, all the available sequences were subdivided further based on their vertebrate host (Table 5). The majority of *A. phagocytophilum* samples belonged to ecotype I or II (Table 5). Ecotype I was isolated significantly more often from cattle, dogs, hedgehogs, horses, mouflons, red deer, sheep, and humans, while ecotype II was isolated significantly more often from roe deer. *Anaplasma phagocytophilum* from wood mouse was identical to the *groEL* sequence found in the engorged *I. trianguliceps.* Both these samples belonged to ecotype III. Likewise, 18 European isolates from rodents, and two isolates originating from *I. persulcatus* belonged to ecotype III*.* Four *I. frontalis*, one *I. ricinus* feeding on common blackbirds, and one spleen from a common blackbird contained *A. phagocytophilum* isolates belonging to ecotype IV. Samples from birds were significantly more often associated with ecotype IV than with other ecotypes (Table 5).

**Table 5:** **Host distributions between ecotypes**

	Ecotype I		Ecotype II		Ecotype III		Ecotype IV		
**Animal**	***All***	***Part***	***All***	***Part***	***All***	***Part***	***All***	***Part***	***Total***
Bird	0	0	0	0	0	0	8*	6*	8
Rodent	0	0	3	0	27*	3*	0	0	30
Hedgehog	59*	7*	0	0	0	0	0	0	59
Cattle	**5**	0	0	0	0	0	0	0	5
Dog	**9**	0	0	0	0	0	0	0	9
Red fox	3	3	0	0	0	0	0	0	3
Goat & sheep	24*	11*	5	0	0	0	0	0	29
Horse	**36**	**14**	0	0	0	0	0	0	36
Moose	1	0	1	0	0	0	0	0	2
Mouflon	18*	14*	0	0	0	0	0	0	18
Red deer	45*	26*	2	0	0	0	0	0	47
Wild boar	3	2*	0	0	0	0	0	0	3
Roe deer	6	3	**66**	**39**	0	0	0	0	72
Human	**34**	0	0	0	0	0	0	0	34
*I. persulcatus*	0	0	**12**	0	3	0	0	0	15
*I. ricinus*	**101**	**23**	68	7	0	0	0	0	169
Deer ked	3	0	**6**	**6**	0	0	0	0	9
**Total**	**347**	**103**	**163**	**52**	**30**	**3**	**8**	**6**	**548**

European samples (All) and Dutch and Belgian samples (Part) are divided in the four ecotypes, which are derived from Figure 1. European samples included the Dutch and Belgian samples. Asterisks* indicate that the *A. phagocytophilum* samples were (partially) derived from ticks feeding on these hosts (Table 3). The most numerous ecotype in bold numerals indicate significant deviations from the hypotheses that ecotypes are evenly represented in that host species (P < 0.05).

**Figure 2:**
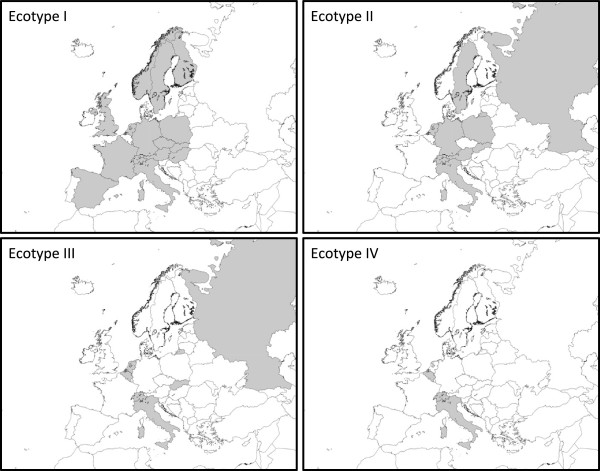
**Geographic distributions of** ***A. phagocytophilum*** **ecotypes in Europe.** Countries in which one or more isolates from an ecotype are found are filled with grey. A country in which an ecotype was not detected or which was not sampled is depicted in white. Data are based on isolates from Table 5. Number of isolates per country can be found in Additional file 3: Table S2.

Ecotype I contained the largest number of distinct hosts, whereas the observed host range of the other three ecotypes was significantly smaller than expected (Table 6), indicating a broad host range for ecotype I and much smaller host ranges for the others. The most abundant host species in ecotype II, III and IV were roe deer, rodents and birds, respectively (Table 5). Visual inspection of the haplotype genealogies within ecotype I indicates a mixture of *A. phagocytophilum* samples of all kind of vertebrate species and *I. ricinus*, indicating transmission of *A. phagocytophilum* between these host species via *I. ricinus*. The presence of ecotypes in European countries were plotted to test whether the clustering could be explained by differences in geographic distribution. All four ecotypes were spread over Europe, and no geographic clustering of the ecotypes was observed (Figure 2).

**Table 6:** **Host distributions within ecotypes**

Hosts (17)	Ecotype I		Ecotype II		Ecotype III		Ecotype IV	
***Sample***	***All***	***Part***	***All***	***Part***	***All***	***Part***	***All***	***Part***
Size	347	103	163	52	30	3	8	6
Observed	14	9	***8***	***3***	***2***	1	***1***	***1***
Expected	14	9	12	8	8	2	5	4

The expected and observed host range of the four ecotypes were calculated for the European samples (All) and Dutch and Belgium samples (Part). Observed: observed number of distinct host species. Expected: expected number of distinct host species given the sample size. Bold italic numerals indicate p-value < 0.025, hence observed host-species richness is significantly less than expectation from the ecotype I. Expected species richness and its p-value were computed using EstimateS (Version 9, R. K. Colwell, http://purl.oclc.org/estimates).

Considerable sequence variation was found between and within ecotypes I, II and III. This prompted us to investigate whether this genetic marker could be used to detect changes in the population dynamics of *A. phagocytophilum* in the wild. The Ewens-Watterson test was performed separately on ecotypes I, II and III to infer the neutrality of the *groEL* marker [49–51]. It was not possible to apply the neutrality test for ecotype IV because only one haplotype was identified in this ecotype (Figure 1). The probability that two randomly chosen samples share the same haplotype (the F-values) agreed to the expectation of neutrality for ecotype III, but not ecotype I and II indicating that a particular haplotype was over-represented within each ecotype. Population expansion was tested using estimates of Fu’s Fs and Tajima’s D. Estimates of Fu’s Fs were significantly negative for ecotype I from the Netherlands and ecotype I from Europe (Table 7). These results demonstrate a large excess of rare genetic variants, over the expected genetic variants under the hypothesis of neutral selection and constant population size.

**Table 7:** **Summary of the population genetic test for** ***A. phagocytophilum***

Cluster	Ecotype I		Ecotype II		Ecotype III		Ecotype IV	
***Sample***	***All***	***Part***	***All***	***Part***	***All***	***Part***	***All***	***Part***
Ewens Watterson	**0.19**	0.10	0.18	0.48	0.18	NA	NA	NA
Tajima’s D	**−1.8**	−0.18	−0.56	−1.45	−0.01	0.00	0.00	0.00
Fu's Fs	**−25,47**	**−8,71**	−1,48	−1,57	1.16	0.00	0.00	0.00

Ewens and Watterson is a test of neutrality. Bold numerals indicate p-values less than 0.05 indicating that a particular haplotype was identified in the ecotype more frequently than the expectation. This test returned Not applicable (NA) when only one haplotype is identified in the sample. Fu’s Fs statistic is a measure of a population expansion based on population genetics. Bold numerals indicate p-values less than 0.05, hence a significant evidence for a population expansion.

## Discussion

This study addressed the circulation of *A. phagocytophilum* in ticks and vertebrates. Our investigations only detected the DNA of this microorganism and not their viability or infectivity. However, previous studies implicate *I. ricinus* ticks as vectors and all investigated animals as potential hosts for *A. phagocytophilum* [4]. Therefore, the inability of a DNA-based detection method to asseverate infectiousness in host species is expected to be a minor issue in this study. In any case, we revealed the widespread circulation of *A. phagocytophilum* in enzootic cycles in Belgium and the Netherlands. In some cases, the infection status of hosts was inferred from the infection rate of infected tick feeding. Vertebrates were considered positive when the infection rate of engorged ticks was significantly higher than that of questing *I. ricinus* (Table 4).

The absence of *A. phagocytophilum* in questing ticks in four out of 17 areas might be attributable to the relatively low number of ticks collected and tested (Table 1). Still, infection rates of *A. phagocytophilum* in questing ticks varied significantly between some geographic locations (Table 1), corroborating results from previous studies from other locations in Europe [4]. Knowledge on the ecological factors driving these differences is of relevance to public and animal health [52], but was not in the scope of this study. Significant differences between the infection rates of *I. ricinus* nymphs and adults were observed as well (Table 2). This may reflect that *A. phagocytophilum* ecotypes I and II cycle mainly between (infected) adults and nymphal *I. ricinus,,* which become infected when feeding on larger vertebrates.

Also, in another study a small but significant proportion of *I. ricinus* larvae were found *A. phagocytophilum*-positive [53]. These larvae may have become positive due to transovarial transmission or due to drop-off from *A. phagocytophilum*-positive hosts after partial feeding and continued to quest as larvae. Transovarial transmission and whether *A. phagocytophilum*-positive larvae can transmit the microorganism to vertebrate hosts needs to be investigated. Together, these findings indicate that all three tick stages should be taken into account when calculating the acarological risk of a given area [12].

In terms of the risk for public health, not only the product of the density of questing ticks and their infection rate defines high or low risk areas, but also the zoonotic potential of the microorganism should be taken into account [12]. The identification of four different *A. phagocytophilum* ecotypes (Figures 1 and 2) with significantly different host ranges and zoonotic potential supports this. A significant correlation between the genetic clustering of *groEL* sequences and different host ranges was found (Tables 5 and 6). Further genetic subclustering within ecotypes I, II and IV was also observed (Additional file 2: Figure S1). These subclusters could not be statistically linked to a further restriction in host ranges or to limitations in geographic distributions (not shown), probably due to lack of resolution in the *groEL locus,* and due to the limitations in the number and origin of the used samples, particularly of rodents and birds.

Clustering of *A. phagocytophilum* isolates can also be achieved using other genetic loci, such as the *ankA* gene, which is presumably involved in host-specific adaptation [21, 54]. Combining several genetic loci, such as *groEL* and *AnkA*, in future analyses could reveal more refined host ranges, especially within ecotype I. Recently, a multilocus sequence typing scheme for *A. phagocytophilum* was presented, which was shown to be informative concerning host species, geographic distribution, and zoonotic potential [54]. The advent of this standardized multilocus sequence typing scheme and a freely available molecular epidemiological database (http://pubmlst.org/aphagocytophilum/) will facilitate more elaborate analyses in the future.

Ecotype I had the broadest host range, but lacked birds and rodents, indicating that the latter two do not contribute directly to the transmission cycle. The generalist feeding behavior of *I. ricinus* nymphs and adults probably facilitates the continuous exchange of ecotype I between the different vertebrate species. All human isolates on the *groEL*-gene from Genbank and the literature [38–42] belong to ecotype I, demonstrating that members of this ecotype are zoonotic. Hence, ecotype I is the most plausible cause of infection regarding the one case of Human Granulocytic Anaplasmosis (HGA) in the Netherlands reported in 1999 [55]. Whether all or only a subset of the members of ecotype I are zoonotic remains to be examined [56].

In this study, ecotype II was found in roe deer, *I. ricinus*, and deer keds (Table 5). Therefore, ecotype II may circulate between roe deer via *I. ricinus*, or deer keds, or both. Whether deer keds may act as a host specific vector for ecotype II remains to be investigated [57, 58]. When the generalist tick *I. ricinus* would transmit ecotype II, then the observed host specificity might be attributed to *A. phagocytophilum* and the possibility that the vectors play a role in host specificity could be excluded. Only three isolates belonging to ecotype III were found in this study. One isolate was found in the spleen of a wood mouse. The kidney, liver and ear of this rodent were all *A. phagocytophilum*-positive, indicating a systemic infection of this wood mouse with *A. phagocytophilum.* Two isolates were found in two different tick species, *I. trianguliceps* and *I. ricinus,* feeding on one wood mouse. Ecotype III was not found in questing *I. ricinus* or in any other wildlife, except rodents (Table 5). Our finding supports the notion that ecotype III might be adapted to a life cycle involving exclusively some rodent species and a rodent specific vector, such as *I. trianguliceps* [18, 23, 59, 60]. Ecotype IV is most likely associated with one or more bird species, but not with other vertebrates. Ecotype IV was not found or in any other animal species. As ecotype IV was not found in questing *I. ricinus* either*,* it might be adapted to a life cycle involving exclusively birds and a bird-specific vector, such as *I. frontalis.* Although *A. phagocytophilum* was not detected in questing *D. reticulatus* or *I. arboricola* ticks, their role in the transmission of one or more *A. phagocytophilum* ecotypes cannot be excluded due to the relatively low numbers of ticks tested [61]. Although many ticks and animal samples have been included in this study, some animal species, particularly birds, rodents and carnivores, and some geographical locations (Figure 2) are underrepresented. Future studies should include broader and randomized sampling strategies.

Before the considerable sequence variation between and within ecotype I and II in the *groEL* gene (Figure 1) could be used to address their population dynamics, several statistical tests were performed to address the neutrality of this genetic marker. The Ewens-Watterson test detected significant departure from neutrality for ecotype I. This outcome indicated that a particular haplotype was identified in ecotype I more frequently than the neutral expectation, indicating that this haplotype is under positive selection. Fu’s Fs statistic detected genetic traces of demographic changes for ecotype I in the Netherlands and Belgium. Fu’s Fs is more sensitive than Tajima’s D to an excess of rare genetic variants in the samples [62], and this has proved to be true for our datasets (Table 7). The increase in ecotype I population sizes might have occurred through an increase in either the population of ixodid ticks, or in the vertebrate host species, or in both [9, 11].

## Conclusions

In conclusion, we identify the *groEL* gene as a suitable marker to discriminate between *A. phagocytophilum* ecotypes. These ecotypes can be linked to distinct host ranges. Furthermore, all three ecotypes have enzootic cycles in the Netherlands and Belgium. In these countries, ecotype I is expanding. This is probably caused by the increase in abundance (and activity) of their vertebrate hosts and vectors. Based on the analyses of the *groEL* marker, we infer that: 1. Ecotype I has the highest zoonotic potential, and 2. the acarological risk of exposure to *A. phagocytophilum* ecotype I has been increasing in time. However, future studies concerning the evolution, population dynamics, and ecology of naturally occurring *A. phagocytophilum* will shed light on identifying risks for public health.

## Electronic supplementary material

Additional file 1: Table S1: DNA sequences and epidemiological data used for this study. (XLSX 53 KB)

Additional file 2: Figure S1: Phylogenetic relationship of *A. phagocytophilum.* Phylogenetic analyses of *groEL* sequences from all *A. phagocytophilum* samples (Table 5) were performed as described in the Methods section. Roman numerals label the four ecotypes. (PDF 41 KB)

Additional file 3: Table S2: Geographic distributions of *A. phagocytophilum* ecotypes in Europe. Number of isolates per country. Data are based on isolates from Table 3. (DOC 36 KB)
