# Supplementary figures and images for: Circulation of four Anaplasma phagocytophilum ecotypes in Europe
Source: Parasit Vectors. 2014 Aug 15;7:365. doi: 10.1186/1756-3305-7-365 (PMC4153903; doi:10.1186/1756-3305-7-365)

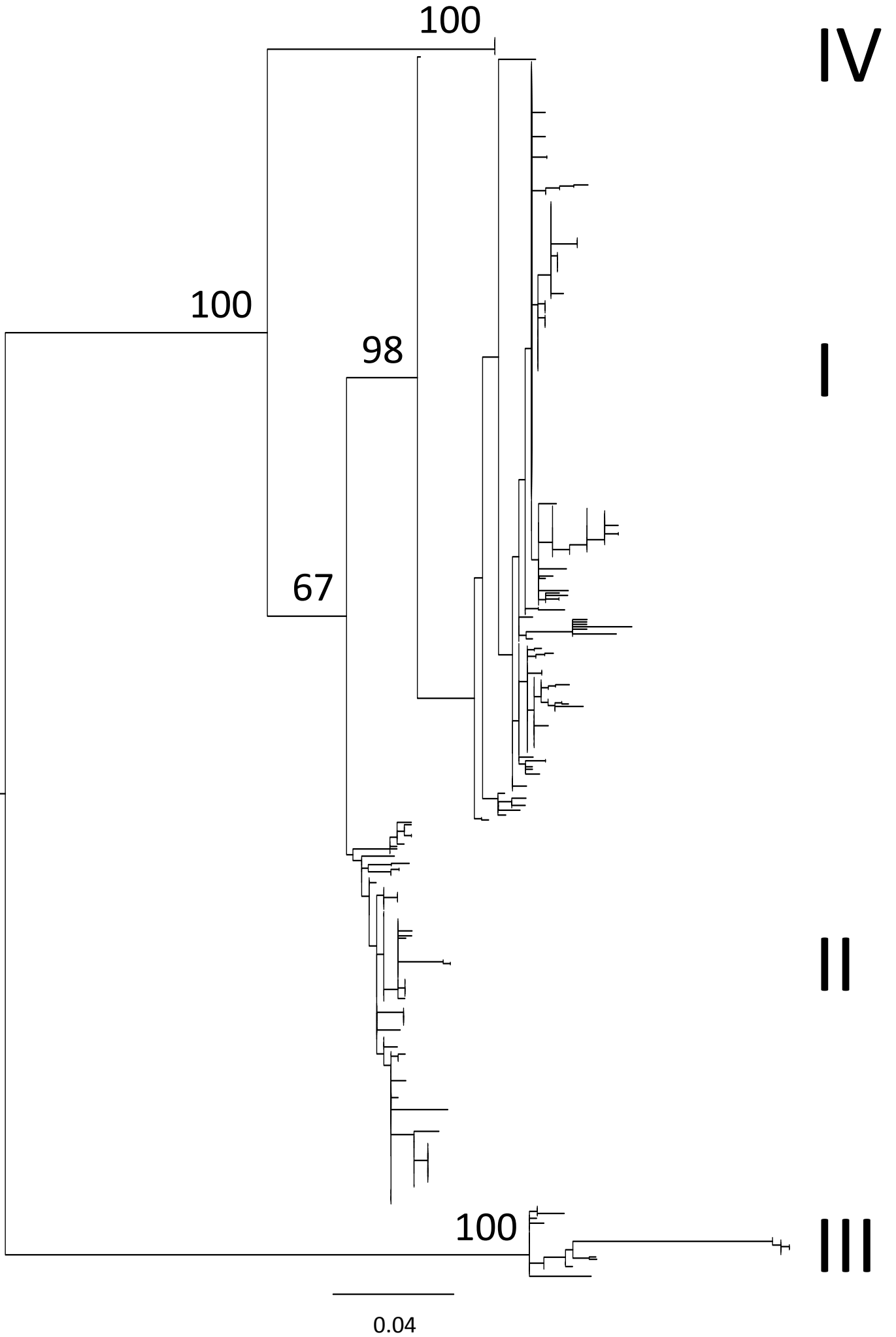

Supplement: Supplementary file 2 — Additional file 2: Figure S1: Phylogenetic relationship of A. phagocytophilum. Phylogenetic analyses of groEL sequences from all A. phagocytophilum samples (Table 5) were performed as described in the Methods section. Roman numerals label the four ecotypes. (PDF 41 KB) [file 13071_2014_1562_MOESM2_ESM.pdf]
