# Supplementary material for: Circulation of four Anaplasma phagocytophilum ecotypes in Europe
Source: Parasit Vectors. 2014 Aug 15;7:365. doi: 10.1186/1756-3305-7-365 (PMC4153903; doi:10.1186/1756-3305-7-365)
Supplement: Supplementary file 3 — Additional file 3: Table S2: Geographic distributions of A. phagocytophilum ecotypes in Europe. Number of isolates per country. Data are based on isolates from Table 3. (DOC 36 KB) [file 13071_2014_1562_MOESM3_ESM.doc]

| **Cluster** | **Ecotype I** | **Ecotype II** | **Ecotype III** | **Ecotype IV** |
| --- | --- | --- | --- | --- |
| Austria | 16 | 11 |  |  |
| Belgium | 1 |  | 1 | 6 |
| Czech Republic | 2 | 1 |  |  |
| Finland | 1 |  |  |  |
| France | 5 |  |  |  |
| Germany | 42 | 23 |  |  |
| Hungary | 93 |  |  |  |
| Italy | 9 | 15 | 3 | 1 |
| Luxembourg | 2 |  |  |  |
| Netherlands | 103 | 24 | 2 |  |
| Norway | 2 | 52 |  |  |
| Poland | 3 |  |  |  |
| Russia |  | 9 | 13 |  |
| Slovakia | 4 | 13 | 10 |  |
| Slovenia | 44 | 1 |  |  |
| Spain | 11 | 8 |  |  |
| Sweden | 3 |  |  |  |
| Switzerland | 4 | 1 | 1 | 1 |
| United Kingdom | 2 | 5 |  |  |
